# Role of HIV Serostatus Communication on Frequent HIV Testing and Self-Testing Among Men Who Have Sex With Men Who Seek Sexual Partners on the Internet in Zhejiang, China: Cross-Sectional Study

**DOI:** 10.2196/57244

**Published:** 2024-11-14

**Authors:** Wanjun Chen, Lin Chen, Zhikan Ni, Lin He, Xiaohong Pan

**Affiliations:** 1 Department of AIDS and STD Prevention and Control Zhejiang Provincial Center for Disease Control and Prevention Hangzhou, Zhejiang China; 2 Department of Infectious Diseases Prevention and Control Yiwu Center for Disease Control and Prevention Yiwu, Zhejiang China

**Keywords:** human immunodeficiency virus, HIV, men who have sex with men, HIV serostatus communication, HIV testing, HIV self-testing

## Abstract

**Background:**

Men who have sex with men (MSM) are increasingly using the internet to meet casual sexual partners. Those who do are at higher risk of sexually transmitted diseases. However, little is known about the rates and associations of frequent HIV testing and self-testing among such MSM.

**Objective:**

We aimed to examine HIV serostatus communication and perceptions regarding the HIV infection risk of internet-based partners, along with their associations with frequent HIV testing and self-testing.

**Methods:**

A cross-sectional study was conducted between May 2018 and April 2019 in Zhejiang Province, China. The study participants were assigned male at birth, were aged 18 years or older, had had casual sex with another male found through the internet in the last 6 months, and were HIV-negative. Information was obtained on HIV-testing behavior, along with demographic characteristics, HIV-related knowledge, internet-based behaviors, sexual behaviors with male partners, HIV serostatus communication, and perceptions regarding the HIV infection risk of internet-based partners. Uni- and multivariate logistic regression models were used to measure the associations of HIV testing and self-testing.

**Results:**

The study recruited 281 individuals who had sought casual sexual partners through the internet during the previous 6 months. Of the participants, 61.9% (174/281) reported frequent HIV testing (twice or more frequently) and 50.9% (119/234; 47 with missing values) reported frequent HIV self-testing. MSM who always or usually communicated about the HIV serostatus of internet-based partners in the previous 6 months had 3.12 (95% CI 1.76-5.52) and 2.45 (95% CI 1.42-4.22) times higher odds of being frequently tested or self-tested for HIV, respectively, compared with those who communicated about this issue minimally or not at all.

**Conclusions:**

There remains a need to improve the frequency of HIV testing and self-testing among internet-based MSM. HIV serostatus communication should be improved within the context of social networking applications to promote frequent HIV testing among internet-based MSM, especially for those who communicated about this issue minimally or not at all.

## Introduction

AIDS remains an urgent public health priority worldwide. According to the Joint United Nations Program on HIV/AIDS, there were an estimated 39 million people living with HIV in 2022 worldwide [1]. However, an estimated 40% of people living with HIV worldwide [2], and 32% in China, are unaware of their HIV status [3]. Such people may engage in higher sexual behaviors associated with (sexually transmitted infections [STI], HIV, etc) risk than those who are aware of their HIV serostatus [4], and in fact account for 60%-80% of all new HIV transmissions [5]. Although much effort has been expended on identifying HIV-infected cases through facility-based testing referring to the testing of voluntary counseling and testing (VCT) and provider-initiated testing and counseling provided in clinical settings, there are barriers to such testing, including inconvenient testing systems, fear of social stigmatization, and lack of privacy. For prompt treatment and prevention of onward infection, more HIV testing strategies need to be explored in addition to facility-based testing [2].

Men who have sex with men (MSM) are disproportionately affected by HIV, both in China and globally, and they are a difficult subgroup to reach through facility-based testing [6,7]. HIV self-testing refers to a process in which a person collects their specimen (oral fluid or blood) and then interprets the result of a simple and rapid HIV test when and where they want [8]. In randomized controlled trials, self-testing appears to promote increased frequency of HIV testing, because of its acceptability and feasibility [9,10]. MSM increasingly use the internet to socialize and meet sexual partners. Our cohort study reported that the internet-based sexual behavior in the past 6 months increased during the 6-monthly follow-up from 2018 to 2020 (unpublished). However, studies have observed more high-risk behaviors among internet-based MSM compared with non–internet-based MSM, such as more sex partners and condomless anal intercourse [11,12]. Furthermore, some studies indicate that internet-based MSM are more likely to have STIs [13], including HIV infections [11]. Oppositely, a study showed men were less likely to have unprotected anal sex in partnerships that they initiated on the web compared with those that they initiated offline [14]. Even so, frequent HIV testing is important.

Factors associated with the likelihood of undergoing HIV testing include age [15,16], education [15], HIV knowledge [15,17], living in an urban area [15], adopting a gay sexual identity [15,17], and sexual experience [15-17]. Several factors influence HIV self-testing rates among MSM, including demographics [18,19], HIV knowledge [19], long-term drug use [18], having self-tested friends [20], previous HIV testing [19,20], and certain sexual behaviors, such as high number of sex partners and sexual activity with male commercial sex workers [18,20]. Previous studies have examined HIV testing and self-testing among MSM from either the internet or venues. However, little is known about the frequency of HIV testing and self-testing among internet-based MSM. Targeting MSM who seek partners through the internet should help us to understand the characteristics of at-risk MSM who are potential candidates for internet-based interventions.

This study explored the rates and associations of frequent HIV testing and self-testing among MSM who seek sexual partners on the internet in Zhejiang province, building upon previous research on internet-based behaviors, HIV serostatus communication, and perceptions of the HIV infection risk posed by internet-based partners. Zhejiang province is a prosperous economic region of in China, located on the southeastern coast. Based on annual sentinel surveillance, the HIV prevalence in Zhejiang during 2018-2020 was 5.62% [21]. It is one of the 3 provinces with the highest average number of daily on the web MSM, with 72,212 MSM among the estimated provincial total of 409,108 MSM by using big data from social networking [22]. Therefore, it is essential to conduct a study focusing on internet-based MSM in Zhejiang province. We aim to provide a scientific basis for public health education interventions targeted at internet-based MSM.

## Methods

### Study Participants and Data Collection

A cross-sectional study was conducted between May 2018 and April 2019 in Zhejiang Province, China. The survey used baseline data collected for a cohort study from 2018 to 2020, including a 6-monthly follow-up to monitor new infections among MSM. In total, 731 MSM were recruited at baseline, with the inclusion criteria of assigned male sex at birth; having had sex with another male; age of ≥16 years and being HIV negative. Participants aged 18 years or older, who had casual sex with partners found through the internet in the last 6 months were selected. Individuals who had sought casual sexual partners through the internet in the past 6 months were defined as internet-based MSM. The analysis included a total of 281 participants.

Participants were enrolled using convenience sampling from MSM venues and the internet by volunteers at nongovernmental organization or community-based organizations as well as VCT staff. Participants enrolled from MSM venues were recruited using published posters in VCT clinics and outreach services (eg, bathrooms, bars, and gardens). Participants enrolled from the internet were recruited through advertisements regarding the study in chat groups on Blued (Beijing Blue City Brothers Culture Media), WeChat (Shenzhen Tencent Computer Systems), and Tencent (Shenzhen Tencent Computer Systems). All participants completed an electronic questionnaire by scanning a 2D code.

### Measurements

The questionnaire covered demographic characteristics, experience with HIV testing services, sexual behaviors with male partners, internet-based behaviors, HIV serostatus communication, perceptions of the HIV risk posed by internet-based partners and HIV-related knowledge. Casual sexual behavior was evaluated by asking “Did you have casual sex in the last 6 months with someone you met through the Internet?” Participants who replied “yes” were recruited, even if they had other types of partners. The main outcome measure (number of times one was HIV tested and self-tested) was assessed through the question “How many times have you undergone a HIV test and how many times have you performed an HIV self-test during your lifetime?” For the analysis, the data were transformed into a binary variable (“Tested once or less” or “Tested twice or more”).

Age was categorized as 18-24, 25-34, and ≥35 years. The residence was dichotomized (Zhejiang vs other provinces). Educational attainment was dichotomized (below university vs university and above). Annual income was self-reported and was classified as ≤30,000, 30,000-80,000 and >80,000 Chinese Yuan (approximately ≤US $429, US $429- $1143 and >US $1143).

HIV-related knowledge was assessed using 3 questions: “Are men who have sex with men the group most seriously affected by AIDS in China at present?”; “Does infection with other sexually transmitted diseases (STDs) increase the risk of HIV infection?” and “Does the use of drugs such as rush, methamphetamine, ecstasy, and k powder increase the risk of HIV infection?” Replies of “Yes” were defined as correct and “No” as incorrect. The answers of each respondent were categorized as mostly wrong (2 or more wrong or unknown answers), somewhat wrong (1 wrong or unknown answer) and correct. Sexual behaviors with male partners in the last 6 months were assessed based on the sexual role (only receptive vs ever insertive sex), sex with a regular partner (no vs yes), and sex with a venue-based casual partner (no vs yes).

Internet-based behaviors were evaluated in terms of the login frequency on MSM-specific social networking applications and the viewing of erotic videos. Participants were asked, “How frequently do you log in to MSM-specific social networking applications?” The possible response included: 3-7 times a week, 1-2 times a week, once a week, and less frequency. For analysis, the option “3-7 times a week” was defined as > 2 times a week, while the remaining responses were classified as ≤ 2 times a week. The participants were also asked, “How frequently do you view erotic videos?” with the same response options.

HIV serostatus communication was evaluated through the question, “In the past 6 months, did you ask your Internet-based partners about their HIV serostatus before sex?” The possible responses were “always or usually” and “little or none.”

The perceived HIV risk of internet-based partners was determined by asking “How do you perceive the HIV risk of Internet-based partners?” with the response options of “very high,” “high,” “average,” and “low.”

### Statistical Analysis

Before data analysis, variables were inspected for missing values. The proportions of missing data ranged from 0% to 3.91% among independent variables. The proportions of missing data were 0% and 16.73% for outcome measures of “frequent HIV testing” and “frequent HIV self-testing.” The demographics of the participants undergoing HIV testing and self-testing are described as frequencies. Uni- and multivariate logistic regression were used to explore the associations of HIV testing and self-testing by complete case analysis. We calculated the crude and adjusted odds ratios (ORs) and their 95% CIs. All factors in the univariate analysis were included in a multivariable regression model, together with age, education, and residence. The final multivariate logistic model was conducted using forward elimination. In addition, we used multiple imputation (5 imputations) to conduct multivariable regression models to assess the sensitivity of results to missing data. We performed these analyses using SPSS software (version 18.0; IBM Corp). We used a mixed effects logistic regression model with a random effect for the respondents ID to assess the sensitivity. We processed the analysis using R software (version 4.4.1; R Foundation for Statistical Computing).

### Ethical Considerations

This study was reviewed and approved by the ethics committee of Zhejiang Provincial Center for Diseases Control and Prevention (2018-033), and all methods were performed in accordance with the relevant guidelines and regulations. Informed consent was obtained electronically from all participants. The raw data did not contain any personal identifying information that can be linked to particular individuals and was anonymized before its use. Participants were given a gift (body shampoo or facial cleanser) worth 30 Chinese Yuan (approximately US $5).

## Results

### Demographics

In total, 281 internet-based MSM were enrolled, among whom 93.6% (262/280) reported having sought sexual partners through MSM-specific social networking applications. The demographic characteristics are summarized in Table 1. Most (127/281, 45.2%) were aged 25~34 years, 63.0% (177/281) had a university or higher education and 59.8% (168/281) were native to Zhejiang Province. Nearly half (126/281, 44.8%) of the respondents had annual incomes between 30,000 and 80,000 RMB and most (67.3%, 189/281) reported their sexual orientation as gay.

Among participants little or none communicating about the HIV serostatus of the internet-based partner before sex, 52.1% (73/140) of the participants were 25-34 years old which is higher than that among participants always or usually communicating about the HIV serostatus (Table S1 in Multimedia Appendix 1). In total, 22.2% (30/140) of the participants little or none communicating about the HIV serostatus of the internet-based partner before sex acquired the average or low perspective of HIV-infected risk toward internet-based partners, higher than that among participants always or usually communicating about the HIV serostatus.

**Table 1:** Demographic characteristics of the participants and the proportions undergoing frequent HIV testing and self-testing among internet-based MSM in Zhejiang Province, May 2018-April 2019.

Variables		Values (N=281), n (%)
**Age (years)**		
	18-24	94 (33.5)
	25-34	127 (45.2)
	≥ 35	60 (21.4)
**Education**		
	Below university level	104 (37)
	Above university level	177 (63)
**Residence**		
	Zhejiang	168 (59.8)
	Other provinces	113 (40.2)
**Yearly income (RMB; 1 RMB=US $0.14)**		
	≤30,000	68 (24.2)
	30,000-80,000	126 (44.8)
	>80,000	87 (31)
**Sexual orientation**		
	Gay	189 (67.3)
	Bisexual	92 (32.7)
**Frequent HIV testing**		
	No	107 (38.1)
	Yes	174 (61.9)
**Frequent HIV self-testing**		
	No	115 (49.1)
	Yes	119 (50.9)
	Missing	47 (0.2)

### Rates of Frequent HIV Testing and Self-Testing Among Internet-Based MSM

Of the 281 participants, 174 (61.9%) reported frequent HIV testing. Of the 234 participants who reported the information of self-testing, 50.9% (n=119) underwent frequent HIV self-testing.

### Factors Associated With Frequent HIV Testing

#### Overview

In the univariate analysis, communication about the HIV serostatus of the internet-based partner before sex and age were significantly associated with frequent HIV testing. Respondents who always or usually communicated about HIV serostatus were more likely to be tested frequently compared with those who little or none communicated (crude OR 2.55, 95% CI 1.54-4.21). The respective ORs of frequent HIV testing were 1.82 (95% CI 1.06-3.14) and 3.00 (95% CI 1.47-6.11) times higher in participants aged 25-34 years and ≥35 years compared with those aged 18-24 years. In the multivariate analysis, significant associations of communication about the HIV serostatus of an internet-based partner before sex (adjusted OR 3.12, 95% CI 1.76-5.52), an age of 25-34 years (adjusted OR 2.71, 95% CI 1.44-5.09), and an age of ≥ 35 years (adjusted OR 4.92, 95% CI 2.19-11.06), consistent condom use with internet-based partners (adjusted OR 1.88, 95% CI 1.04-3.39) remained significantly associated with frequent HIV testing (Table 2). Multiple imputation for missing data and mixed effects logistic regression model generated similar results (Tables S2 and S3 in Multimedia Appendix 1).

**Table 2:** Univariate and multivariate regression analysis of factors associated with frequent HIV testing among internet-based MSM in Zhejiang Province, May 2018-April 2019.

Variables		Total (N=281), n (%)	Frequent HIV testing (n=174), n (%)	Crude OR^a^ (95% CI)	Adjusted OR^a^ (95% CI)
**Age (years)**					
	18-24	94 (33.5)	47 (27)	Reference	Reference
	25-34	127 (45.2)	82 (47.1)	1.82 (1.06-3.14)^c^	2.71 (1.44-5.09)^c^
	≥ 35	60 (21.4)	45 (25.9)	3.00 (1.47-6.11)^c^	4.92 (2.19-11.06)^d^
**Education**					
	< University	104 (37)	66 (37.9)	Reference	—^b^
	≥ University	177 (63)	108 (62.1)	0.90 (0.55-1.49)	—^b^
**Residence**					
	Zhejiang	168 (59.8)	100 (57.5)	Reference	—^b^
	Other provinces	113 (40.2)	74 (42.5)	1.29 (0.79-2.12)	—^b^
**Knowledge of HIV**					
	Mostly wrong or unknown	38 (13.5)	23 (13.2)	Reference	—^b^
	Somewhat wrong or unknown	61 (21.7)	30 (17.2)	1.29 (0.63-2.66)	—^b^
	Correct	182 (64.8)	121 (69.5)	0.63 (0.28-1.44)	—^b^
**Sexual role**					
	Receptive sex	69 (24.6)	39 (22.4)	Reference	—^b^
	Insertive sex or both	212 (75.4)	135 (77.6)	1.35 (0.78-2.34)	—^b^
**Regular partner**					
	No	39 (13.9)	21 (12.1)	Reference	—^b^
	Yes	242 (86.1)	153 (87.9)	1.47 (0.75-2.91)	—^b^
**Venue-based casual partners**					
	No	214 (77.8)	133 (78.2)	Reference	—^b^
	Yes	61 (22.2)	37 (21.8)	0.94 (0.52-1.68)	—^b^
	Missing data	—^b^	—^b^	—^b^	—^b^
**Frequency of dates with internet-based partners per week**					
	≤ 2 times	97 (34.5)	58 (33.3)	Reference	—^b^
	> 2 times	184 (65.5)	116 (66.7)	1.15 (0.69-1.90)	—^b^
**Frequency of viewing erotic videos per week**					
	≤ 2 times	217 (77.2)	132 (75.9)	Reference	—^b^
	> 2 times	64 (22.8)	42 (24.1)	1.23 (0.69-2.20)	—^b^
**Communication about the HIV serostatus of internet-based partner before sex**					
	Little or none	140 (51.1)	71 (42.3)	Reference	Reference
	Always or usually	134 (48.9)	97 (57.7)	2.55 (1.54-4.21)^d^	3.12 (1.76-5.52)^d^
	Missing data	—^b^	—^b^	—^b^	—^b^
**Perceived HIV-infection risk of internet-based partners**					
	Average or low	44 (16.3)	24 (14.3)	Reference	—^b^
	High	137 (50.7)	81 (48.2)	2.02 (0.96-4.27)^c^	—^b^
	Very high	89 (33.0)	63 (37.5)	1.21 (0.61-2.39)	—^b^
	Missing data	—^b^	—^b^	—^b^	—^b^
**HIV education from social networking applications**					
	No	78 (27.8)	55 (31.6)	Reference	—^b^
	Yes	203 (72.2)	119 (68.4)	0.59 (0.34-1.04)^e^	—^b^
**Condom use with internet-based partners**					
	Inconsistently	84 (30.7)	45 (26.5)	Reference	Reference
	Consistently	190 (69.3)	125 (73.5)	1.67 (0.99-2.81)^e^	1.88 (1.04-3.39)^c^
	Missing data	—^b^	—^b^	—^b^	—^b^

^a^OR: odds ratio.

^b^—: not applicable.

^c^*P*<0.05.

^d^*P*<0.001.

^e^*P*<0.1.

#### Factors Associated With Frequent HIV Self-Testing

There was a significant relationship between communication about the HIV serostatus of internet-based partners before sex and frequent HIV self-testing in the uni- and multivariate analyses. The adjusted OR was 2.45 times (95% CI 1.42-4.22) higher for the participants who always or usually communicated than those who little or none did. None of the other variables were significant (Table 3). Multiple imputation for missing data and mixed effects logistic regression model generated similar results (Tables S3 and S4 in Multimedia Appendix 1).

**Table 3:** Univariate and multivariate regression analysis of factors associated with frequent HIV self-testing among internet-based MSM in Zhejiang Province, May 2018-April 2019.

Variables			Total (N=234), n (%)		Frequent HIV self-testing (n=119), n (%)		Crude OR^a^ (95% CI)		Adjusted OR^a^ (95% CI)
**Age (years)**									
	18-24	73 (31.2)		38 (31.9)		Reference		—^b^	
	25-34	108 (46.2)		58 (48.7)		1.07 (0.59-1.94)		—^b^	
	≥35	53 (22.6)		23 (19.3)		0.71 (0.35-1.44)		—^b^	
**Education**									
	<University	95 (40.6)		42 (35.3)		Reference		—^b^	
	≥University	139 (59.4)		77 (64.7)		1.57 (0.93-2.66)^c^		—^b^	
**Registered residence**									
	Zhejiang	135 (57.7)		67 (56.3)		Reference		—^b^	
	Other provinces	99 (42.3)		52 (43.7)		1.12 (0.67-1.89)		—^b^	
**Knowledge of HIV**									
	Mostly wrong or unknown	32 (13.7)		15 (12.6)		Reference		—^b^	
	Somewhat wrong or unknown	51 (21.8)		31 (26.1)		1.06 (0.49-2.28)		—^b^	
	Correct	151 (64.5)		73 (61.3)		1.76 (0.72-4.29)		—^b^	
**Sexual role**									
	Receptive sex	57 (24.4)		25 (21.0)		Reference		—^b^	
	Insertive sex or both	177 (75.6)		94 (79.0)		1.45 (0.80-2.64)		—^b^	
**Regular partner**									
	No	30 (12.8)		18 (15.1)		Reference		—^b^	
	Yes	204 (87.2)		101 (84.9)		0.65 (0.30-1.43)		—^b^	
**Venue-based casual partner**									
	No	179 (77.5)		89 (75.4)		Reference		—^b^	
	Yes	52 (22.5)		29 (24.6)		1.28 (0.69-2.37)		—^b^	
	Missing data	—^b^		—^b^		—^b^		—^b^	
**Frequency dating internet-based partners per week**									
	≤2 times	84 (35.9)		47 (39.5)		Reference		—^b^	
	>2 times	150 (64.1)		72 (60.5)		0.73 (0.43-1.24)		—^b^	
**Frequency viewing erotic videos per week**									
	≤2 times	180 (76.9)		89 (74.8)		Reference		—^b^	
	>2 times	54 (23.1)		30 (25.2)		1.28 (0.69-2.36)		—^b^	
**Communication about the HIV serostatus of internet-based partner before sex**									
	Little or none	108 (47)		43 (37.1)		Reference		Reference	
	Always or usually	122 (53)		73 (62.9)		2.25 (1.33-3.82)^d^		2.45 (1.42–4.22)^e^	
	Missing data	—^b^		—^b^		—^b^		—^b^	
**Perceived HIV-infection risk of internet-based partners**									
	Average or low	34 (15)		17 (14.7)		Reference		—^b^	
	High	113 (49.8)		55 (47.4)		1.22 (0.55-2.73)		—^b^	
	Very high	80 (35.2)		44 (37.9)		0.95 (0.44-2.04)		—^b^	
	Missing data	—^b^		—^b^		—^b^		—^b^	
**HIV education from social networking applications**									
	No	165 (70.5)		87 (73.1)		Reference		—^b^	
	Yes	69 (29.5)		32 (26.9)		1.29 (0.73-2.27)		—^b^	
**Condom use with internet-based partners**									
	Inconsistently	69 (30.1)		28 (23.9)		Reference		—^b^	
	Consistently	160 (69.9)		89 (76.1)		1.84 (1.04-3.26)^d^		—^b^	
	Missing data	—^b^		—^b^		—^b^		—^b^	

^a^OR: odds ratio.

^b^—: not applicable.

^c^*P*<.10.

^d^*P*<.05.

^e^*P*<.001.

## Discussion

### Principal Findings

We conducted a cross-sectional survey of internet-based MSM in Zhejiang Province to describe the rates and associations of frequent HIV testing and self-testing. Overall, 61.9% of internet-based MSM underwent frequent HIV testing and 50.9% performed frequent self-testing. Communication about HIV serostatus of the internet-based partner before sex was significantly associated with frequent HIV testing and self-testing among internet-based MSM. Our results add to the literature on the associations among internet-based behaviors, HIV serostatus communication, and perceived HIV infection risk of internet-based partners with the frequency of HIV testing and self-testing.

HIV testing and self-testing were not performed by approximately 40% and 50% respectively of the internet-based MSM. In a large STI clinic in the Netherlands, the proportion was 55.2% [23], while it was 57.8% among black MSM in the United States [24], suggesting that there is room for improvement in testing frequency. The World Health Organization recommends HIV self-testing as an additional approach because of its convenience and confidentiality. The US Centers for Disease Control and Prevention recommends at least annual HIV tests for high-risk populations (eg, MSM), with some experts suggesting that more frequent (eg, every 3-6 months) testing benefits individuals at elevated HIV risk [25]. Increasing the acceptability and feasibility of HIV self-testing for this population is considered highly desirable [26]. However, HIV self-testing often lacks face-to-face pre- or posttest consulting, confirmatory testing, and subsequent referral to specialist care. Therefore, it is important to emphasize the benefits and limitations (eg, window period) of self-testing [27,28] when applying this approach.

Of our participants, more than half reported communicating about HIV serostatus of an internet-based partner before sex. That behavior was associated with increased odds of frequent HIV testing and self-testing. Determining HIV serostatus allows individuals to make informed decisions about how to engage in sexual behaviors. However, studies have found that most disclosures occur between steady partners [29]; only a small proportion of men were informed of the HIV serostatus of their most recent casual male partner [30]. Therefore, it is important for MSM to actively communicate their HIV serostatus with internet-based partners. However, merely knowing the HIV serostatus of sexual partners is insufficient to reduce the risk of HIV transmission; knowledge of one’s own HIV status is also important. Unfortunately, MSM who test for HIV infrequently might misperceive their positive HIV status as HIV-negative. Therefore, HIV serostatus communication and frequent testing are both urgent issues for MSM.

An increasing number of MSM use the internet to find partners, especially MSM-specific social networking applications [31], making them an effective target for MSM interventions [11]. Blued, the largest gay male–oriented social media platform and geosocial networking mobile app in China had approximately 27 million registered users and 12 million monthly users in China as of 2016 [32]. Other MSM-specific social networking apps, such as Jack’d and Grindr, have also been downloaded by millions of people, offering new ways for MSM to engage in peer-to-peer communication [33]. Many of the apps require information regarding HIV disclosure, basic demographics, and preferred sexual behaviors in the personal profile. Studies have shown that only 55% of the users report using the HIV disclosure option in the US [34], and in India, few individuals disclose their HIV status on their profiles because of the stigma in the culture [35]. Status disclosure is often easier through the internet than face-to-face. However, doubts persist regarding the authenticity of the results uploaded on apps by the users themselves. It is recommended that apps implement rules to ensure the security and the veracity of disclosures. Apps can also encourage users to regularly update the HIV test results (eg, every 3 months). In general, dating apps are valuable targets for intervention promoting HIV serostatus communication.

### Limitations

This study had several important limitations. First, because of its cross-sectional design, we cannot infer causality in the relationships of HIV serostatus communication with frequent HIV testing and self-testing. Second, participants were recruited through convenience sampling from MSM venues and the internet, and might not be representative of the entire MSM population. Third, information bias, especially recall bias, was possible due to the reliance on self-report methods. Finally, some important factors that could affect the testing behavior of the targeted population were not included in the questionnaire, such as social stigma, cultural diversity, and psychological factors. Future studies should address these limitations, and aim to replicate our findings in both similar populations and different sociocultural groups.

### Conclusions

There is a need to improve the frequency of HIV testing and self-testing among internet-based MSM. Communication about the HIV serostatus of the internet-based partner before sex was significantly associated with frequent HIV testing and self-testing among internet-based MSM. HIV serostatus communication should be improved within the context of social networking apps to promote HIV frequent HIV testing among internet-based MSM.

## Appendix

Multimedia Appendix 1 Characteristics of participants by “Communication about the HIV serostatus of Internet-based partner before sex” and sensitivity analyses of multivariate regression.

## Abbreviations

MSM: men who have sex with men
OR: odds ratio
STD: sexually transmitted disease
STI: sexually transmitted infection
VCT: voluntary counseling and testing

## Data Availability

The datasets used and analyzed are available from the corresponding author (Xiaohong Pan, Email: xhpan@cdc.zj.cn) upon reasonable request.
