# Supplementary material for: Role of HIV Serostatus Communication on Frequent HIV Testing and Self-Testing Among Men Who Have Sex With Men Who Seek Sexual Partners on the Internet in Zhejiang, China: Cross-Sectional Study
Source: JMIR Form Res. 2024 Nov 14;8:e57244. doi: 10.2196/57244 (PMC11605257; doi:10.2196/57244)
Supplement: Multimedia Appendix 1 [file formative_v8i1e57244_app1.docx]

Table S1 Characteristics of participants by “Communication about the HIV serostatus of Internet-based partner before sex” among Internet-based MSM in Zhejiang Province, May 2018 –April 2019

| Variables | | Little/none (n=140) |  |  | Always/usually (n=134) | χ^2^ | *P* value |
| --- | --- | --- | --- | --- | --- | --- | --- |
| **Age (year) , n (%)** | |  |  |  |  | n, % |  |
|  | 18~24 | 37 (26.4) |  |  | 54 (40.3) | 6.968 | 0.03 |
|  | 25~34 | 73 (52.1) |  |  | 51 (38.1) |  |  |
|  | ≥ 35 | 30 (21.4) |  |  | 29 (21.6) |  |  |
| **Education, n (%)** | |  |  |  |  |  |  |
|  | < University | 52 (37.1) |  |  | 51 (38.1) | 0.025 | 0.88 |
|  | ≥ University | 88 (62.9) |  |  | 83 (61.9) |  |  |
| **Registered residence, n (%)** | |  |  |  |  |  |  |
|  | Zhejiang | 77 (55.0) |  |  | 86 (64.2) | 2.394 | 0.12 |
|  | Other provinces | 63 (45.0) |  |  | 48 (35.8) |  |  |
| **Sexual role, n (%)** | |  |  |  |  |  |  |
|  | Receptive sex | 32 (22.9) |  |  | 36 (26.9) | 0.590 | 0.44 |
|  | Insertive sex/both | 108 (77.1) |  |  | 98 (73.1) |  |  |
| **Knowledge of HIV, n (%)** | |  |  |  |  |  |  |
|  | Mostly wrong/unknown | 24 (17.1) |  |  | 13 (9.7) | 4.425 | 0.11 |
|  | Somewhat wrong/unknown | 33 (23.6) |  |  | 27 (20.1) |  |  |
|  | Correct | 83 (59.3) |  |  | 94 (70.1) |  |  |
| **Regular partner, n (%)** | |  |  |  |  |  |  |
|  | No | 16 (11.4) |  |  | 23 (17.2) | 1.845 | 0.17 |
|  | Yes | 124 (88.6) |  |  | 111 (82.8) |  |  |
| **Venue-based casual partners, n (%)** | |  |  |  |  |  |  |
|  | No | 111 (79.3) |  |  | 99 (75.6) | 0.535 | 0.47 |
|  | Yes | 29 (20.7) |  |  | 32 (24.4) |  |  |
|  | Missing |  |  |  |  |  |  |
| **Frequency of dates with Internet-based partners/week, n (%)** | |  |  |  |  |  |  |
|  | ≤ 2 times | 40 (28.6) |  |  | 51 (38.1) | 2.779 | 0.10 |
|  | > 2 times | 100 (71.4) |  |  | 83 (61.9) |  |  |
| **Frequency of viewing erotic video/week, n (%)** | |  |  |  |  |  |  |
|  | ≤ 2 time | 113 (80.7) |  |  | 97 (72.4) | 2.651 | 0.10 |
|  | > 2 times | 27 (19.3) |  |  | 37 (27.6) |  |  |
| **Perceived HIV infected risk of Internet-based partners, n (%)** | |  |  |  |  |  |  |
|  | Average/low | 30 (22.2) |  |  | 11 (8.5) | 12.041 | 0.002 |
|  | High | 69 (51.1) |  |  | 65 (50.4) |  |  |
|  | Very high | 36 (26.7) |  |  | 53 (41.1) |  |  |
|  | Missing |  |  |  |  |  |  |
| **HIV education from social networking applications, n (%)** | |  |  |  |  |  |  |
|  | No | 36 (25.7) |  |  | 41 (30.6) | 0.808 | 0.37 |
|  | Yes | 104 (74.3) |  |  | 93 (69.4) |  |  |

Table S2 Multivariate regression analyses of factors associated with frequent HIV testing after multiple imputation for missing data among Internet-based MSM in Zhejiang Province, May 2018 –April 2019

| Variables | | aOR (95% CI) | | | | |
| --- | --- | --- | --- | --- | --- | --- |
|  |  | MI 1 | MI 2 | MI 3 | MI 4 | MI 5 |
| **Age (y), n (%)** | |  |  |  |  |  |
|  | 18~24 |  |  |  |  |  |
|  | 25~34 | 2.32 (1.30-4.14)^a^ | 2.63 (1.27-4.03)^a^ | 2.38 (1.33-4.27)^a^ | 2.47 (1.37-4.47)^a^ | 2.31 (1.29-4.13)^a^ |
|  | ≥ 35 | 3.65 (1.73-7.68)^b^ | 3.57 (1.70-7.49)^b^ | 3.71 (1.76-7.84)^b^ | 4.21 (1.96-9.08)^b^ | 3.67 (1.74-7.74)^b^ |
| **Communication about the HIV serostatus of Internet-based partner before sex, n (%)** | |  |  |  |  |  |
|  | Little/none |  |  |  |  |  |
|  | Always/usually | 3.04 (1.79-5.17)^b^ | 2.92 (1.73-4.95)^b^ | 3.10 (1.82-5.29)^b^ | 2.86 (1.67-4.92)^b^ | 3.11 (1.83-5.28)^b^ |
| **Condom use with Internet-based partners, n (%)** | |  |  |  |  |  |
|  | Inconsistently |  |  |  |  |  |
|  | Consistently |  |  |  | 1.81 (1.03-3.17)^a^ |  |

^a^*p*<0.05; ^b^*p*<0.001; MI: multiple imputation

Table S3 Mixed effects logistic regression analyses of factors associated with frequent HIV testing and frequent HIV self-testing mong Internet-based MSM in Zhejiang Province, May 2018 –April 2019

| Variables | | Frequent HIV testing | |  | Frequent HIV self-testing | |
| --- | --- | --- | --- | --- | --- | --- |
|  |  | aOR (95% CI) | *P* value |  | aOR (95% CI) | *P* value |
| **Age (y), n (%)** | |  |  |  |  |  |
|  | 18~24 |  |  |  |  |  |
|  | 25~34 | 2.63 (1.37-5.04) | 0.004 |  |  |  |
|  | ≥35 | 4.82 (2.04-11.39) | <0.001 |  |  |  |
| **Communication about the HIV serostatus of Internet-based partner before sex, n (%)** | |  |  |  |  |  |
|  | Little/none |  |  |  |  |  |
|  | Always/usually | 2.97 (1.62-5.45) | <0.001 |  | 2.52 (1.33-3.82) | 0.003 |
| **Condom use with Internet-based partners, n (%)** | |  |  |  |  |  |
|  | Inconsistently |  |  |  |  |  |
|  | Consistently | 1.79 (0.98-3.26) | 0.057 |  |  |  |

Table S4 Multivariate regression analyses of factors associated with frequent HIV self-testing after multiple imputation for missing data among Internet-based MSM in Zhejiang Province, May 2018 –April 2019

| Variables | | aOR (95% CI) | | | | |
| --- | --- | --- | --- | --- | --- | --- |
|  |  | MI 1 | MI 2 | MI 3 | MI 4 | MI 5 |
| **Communication about the HIV serostatus of Internet-based partner before sex, n (%)** | |  |  |  |  |  |
|  | Little/none |  |  |  |  |  |
|  | Always/usually | 2.50 (1.54-4.03)^b^ | 2.02 (1.26-3.26)^b^ | 2.21 (1.37-3.55)^b^ | 2.21 (1.36-3.60)^b^ | 1.96 (1.21-3.16)^b^ |
| **Condom use with Internet-based partners, n (%)** | |  |  |  |  |  |
|  | Inconsistently |  |  |  |  |  |
|  | Consistently |  |  |  | 1.87 (1.10-3.19)^a^ |  |
| **Education** | |  |  |  |  |  |
|  | <University |  |  |  |  |  |
|  | ≥University |  |  |  |  | 1.79 (1.09-2.94)^a^ |

^a^*p*<0.05; ^b^*p*<0.001; MI: multiple imputation
